# Evaluation of the transmission of SARS-CoV-2 through breast milk: a case series

**DOI:** 10.3389/fped.2023.1160790

**Published:** 2023-06-22

**Authors:** Yayoi Murano, Shinya Yamahira, Hiromichi Shoji, Ken Hisata, Takuya Koshizaka, Tomoyuki Nakazawa, Toshiaki Shimizu, Mahbubur Rahman

**Affiliations:** ^1^ Division of Pediatrics, Toshima Hospital, Tokyo, Japan; ^2^ Graduate School of Public Health, St. Luke’s International University, Tokyo, Japan; ^3^ Department of Pediatrics, Juntendo University Faculty of Medicine, Tokyo, Japan; ^4^ Center for Medical Sciences, St. Luke’s International University, Tokyo, Japan

**Keywords:** breastfeeding, mother milk, COVID-19, SARS-CoV-2, pandemic

## Abstract

Whether severe acute respiratory syndrome coronavirus 2 (SARS-CoV-2) is transmitted through breast milk remains controversial. This study aimed to determine the presence of SARS-CoV-2 in breast milk and assess its transmissibility to the child in infancy. Eleven samples were obtained from nine mothers with coronavirus disease 2019 (COVID-19). All but one sample had negative results on a reverse transcription-quantitative polymerase chain reaction. Among nine children, five were diagnosed with COVID-19, including one child whose mother's milk tested positive. Although SARS-CoV-2 RNA was detected in breast milk, its possible transmission via breastfeeding could not be established. Thus, we conclude that the physical attachment between mother and child is a conceivable transmission route.

## 1. Introduction

Severe acute respiratory syndrome coronavirus 2 (SARS-CoV-2) spread globally, resulting in a pandemic. By the end of February 2022, approximately 430 million people had been infected with the virus and 5 million people had died of the disease (1).

Since the beginning of the pandemic, researchers have been investigating whether breast milk can be contaminated by SARS-CoV-2. Mother's milk has nutritional and immunologic benefits, so mothers with coronavirus disease 2019 (COVID-19) are recommended to breastfeed (2). Although most breast milk samples tested negative for SARS-CoV-2 on performing polymerase chain reaction (PCR) (3–6), a few studies have reported the presence of SARS-CoV-2 in breast milk (3, 5, 7, 8). However, these study samples were mainly obtained from puerperal mothers (4, 6, 9, 10). Breastfeeding is recommended for more than 1 year after birth (11), concerns are not only about neonates but also infants, because they are breastfed too. However, only a few studies have assessed infants breastfeeding from mothers with COVID-19 (3); therefore, additional studies are needed to examine whether SARS-CoV-2 transmission to older infants is possible through breast milk. Moreover, methods of milk collection in these studies were either not clearly reported or were inappropriate and hence might be responsible for viral contamination. For example, some samples were collected without the use of face masks (8) or the samples were self-collected (3).

## 2. Methods

Women who were currently breastfeeding their infants and were diagnosed with COVID-19 on reverse transcription-quantitative PCR (RT-qPCR) using nasopharyngeal swab samples were enrolled. Information regarding COVID-19 diagnosis for both mothers and infants was collected from their medical records. Written informed consent was obtained from the mothers.

Milk samples were collected under the guidance of the medical staff; mothers were instructed to wear face masks, milk collectors' hand hygiene was maintained using soap, and mothers' nipples were wiped using cotton with alcohol. The milk samples were immediately stored at −80°C. At the same time, we wiped the nipple with a swab, which we kept in virus transportation media.

RNA was isolated using the QIAamp Viral RNA Mini Kit (QIAGEN, Hilden, Germany) following standard instructions. RT-qPCR was performed using TaqMan Fast Virus 1-Step Master Mix (Thermo Fisher Scientific, Waltham, MA, USA) and the 2019-nCoV primer/probe set (Takara Bio Inc., Shiga, Japan). RT-qPCR was performed in a MicroAmp™ Fast Optical 96-well reaction plate with 20 µl of the mixture per well containing 5 µl of TaqMan Fast Virus 1-Step Master Mix, 4 µl of primer/probe mix, 6 µl of distilled deionized water (Nippon Gene, Tokyo, Japan), and 5 µl of extracted RNA solution. A standard curve was obtained using a healthy mother's milk, which was obtained from a mother who had no history of COVID-19, using the same procedure as described above. The healthy mother's milk was spiked with a positive control RNA mix (2019-nCoV; No. XA0142, Takara Bio Inc.) after RNase inactivation of the milk sample with AVL buffer from the QIAamp Viral RNA Mini Kit. Analysis was performed using Step One Plus™ (Applied Biosystems, Foster City, CA, USA), and the results were analyzed using Step One Plus™ software v2.3. RT-qPCR was conducted under the following thermal cycling conditions: 5 min at 50°C for reverse transcription, 20 s at 95°C for initial denaturation, 45 cycles of 3 s at 95°C for denaturing, 30 s at 60°C for annealing and extension, and hold at 4°C.

This study was approved by the ethical committee of Toshima Hospital.

## 3. Results

### 3.1. Study samples

A total of nine mothers diagnosed with COVID-19 based on SARS-CoV-2 PCR results were included in this study. In total, 11 milk samples were obtained from the participants. Table 1 shows the characteristics of the mothers. In Table 1, maternal age and child age are shown with the case number, followed by the PCR results of the children and maternal history. In mothers diagnosed with COVID-19 before child onset of COVID-19, the period from maternal onset to child onset is included. The following column shows paternal history from the medical records if it was available. In the last line, the date of the breast milk collection has been included. Among the nine infants, five tested positive for COVID-19 on SARS-CoV-2 PCR. Of these five infants with COVID-19, the mothers of two developed COVID-19 symptoms after they did, and their fathers had symptoms before the infants were subjected to the SARS-CoV-2 PCR test.

**Table 1:** Sample characteristics.

Case No.	Mother's age (years)	Child's age (months)	Child PCR	Maternal history	Mother-child contact period before PCR (days)	Paternal history	Date mother's milk was obtained
							Date of onset of symptom
1	21	0	Negative	Cough 1 day before PCR	1		7, 10, Aug 2021
2	35	10	Positive	Fever 5 days before PCR	5		7, 10 July 2021
3	31	7	Positive	Sore throat 2 days after child's onset of symptom	—	Fever 3 days before child onset	7, Dec 2021
4	41	5	Positive	Cough 1 day after child onset of symptom	—	Fever 1 day before child onset	7, Oct 2021
5	34	3	Negative	Fever 4 days before PCR	4		7, Oct 2021
6	33	3	Positive	Fever 3 days before PCR	3	Fever 5 days before child onset	10, Sep 2021
7	32	3	Negative	Performed PCR as father's close contact person	—	Fever 3 days before PCR	9, Jun 2022
8	34	6	Positive	Fatigue 1 day before PCR	1		8, Feb 2022
9	32	6	Negative	Fever a day before PCR	1		7, Apr 2022

### 3.2. Results of RT-qPCR

All samples, except for case 4, tested negative on RT-qPCR. Of three of the tested samples from case 4, two were positive, with Ct values of 34.6 and 36.1 and copy number of 2.48 ± 1.56 copies/µl in the extracted RNA solution (1.06 ± 0.67 copies/µl in the breast milk). We performed RT-qPCR again on this sample and the result was positive. We also performed RT-qPCR with the sample of an RT-qPCR-positive case obtained by wiping the mother's nipples, and the results were negative.

## 4. Discussion

In this study, we tested the breast milk of women with COVID-19 whose offspring were aged 0–10 months. One of the samples had a positive SARS-CoV-2 PCR result but a negative result for the swab sample collected from the nipple skin.

Most previous studies have focused on puerperal mothers (4, 6, 9, 10). However, because the breastfeeding period extends beyond the neonatal period, and the characteristics that might be responsible for this viral transmission through breast milk might change over time (12), we included mothers who had older infants. In addition, in our study, we tested the nipple skin surface of the mothers to examine the possibility of virus transmission through this route.

In the present study, five mother–infant pairs were diagnosed with COVID-19 (cases 2, 3, 4, 6, and 8). Among these five infants, the mothers of two infants developed COVID-19-related symptoms after their infants' diagnosis (cases 3 and 4), and their fathers were the suspected source of infection. Our experience supports the idea that infants are already colonized by SARS-CoV-2 and that breastfeeding brings greater benefit than harm (13). Although the sample size was small, older infants were more likely to have positive results (positive infants were 3, 6, 6, 7, and 10 months old, whereas negative infants were 0, 3, 3, and 6 months old). This difference could be because older children are more likely to be exposed to infected people as well as infection sources. This idea is supported by the opinion that the main route of transmission of SARS-CoV-2 is via infected droplets of caregivers (13). In our study, we used hygiene methods during sample collection to exclude contamination of droplets from the mothers to the breast milk samples. Moreover, the sample obtained from the skin of the mother whose milk had a positive SARS-CoV-2 PCR result showed a negative SARS-CoV-2 PCR result. This suggests that the positive SARS-CoV-2 PCR result of the mother's milk was not due to droplet contamination. In the present study, we found that five of nine infants were infected with COVID-19, and it is more likely that the SARS-CoV-2 infection occurred not via the mother's milk but rather via droplets, as discussed in a previous study (13).

Samples from only mothers with mild-to-moderate symptoms were analyzed herein. A previous study reported that RNAemia results in severe symptoms (14), while another study reported that viral load is associated with COVID-19 severity (15). Because breast milk is produced from blood, more frequent viral contamination may occur in the breast milk of severe patients. However, because breastfeeding is recommended only in mothers who are adequately healthy (5), we believe that our results can provide guidance to most infected women who are breastfeeding their infants.

This study had some limitations. First, the presence of RNA does not indicate infection (16). In a previous study, instances of positive PCR with negative viral cultures have been reported (3). Infection routes were reported to be mainly respiratory and orofecal (17). However, some studies have reported gastrointestinal infection of SARS-CoV-2 (18), and the transmission of infection from breast milk to infants via the gastrointestinal route is a possibility. Second, we did not consider the time of day, which might influence the variation in viral concentrations (12). Finally, we could not conduct an analysis based on samples from the early phase of infection, as the patients were admitted depending on hospital bed availability. In addition, the days that milk samples were obtained were different. However, the period was short between day 7 and day 10.

Because a standard procedure to identify antibodies against SARS-CoV-2 in breast milk is already available, further studies might shed more light on the protective aspects of breast milk against mother-to-infant viral transmission.

## 5. Conclusion

In mothers with COVID-19, virus transmission via breastfeeding is not plausible, and breastfeeding under hygienic conditions is recommended.

## Data Availability

The raw data supporting the conclusions of this article will be made available by the authors, without undue reservation.
